# Gene Discovery and Molecular Marker Development, Based on High-Throughput Transcript Sequencing of *Paspalum dilatatum* Poir

**DOI:** 10.1371/journal.pone.0085050

**Published:** 2014-02-10

**Authors:** Andrea Giordano, Noel O. I. Cogan, Sukhjiwan Kaur, Michelle Drayton, Aidyn Mouradov, Stephen Panter, Gustavo E. Schrauf, John G. Mason, German C. Spangenberg

**Affiliations:** 1 Department of Environment and Primary Industries, AgriBio, Centre for AgriBioscience, Bundoora, Victoria, Australia; 2 Molecular Plant Breeding Cooperative Research Centre, Bundoora, Victoria, Australia; 3 Dairy Futures Cooperative Research Centre, Bundoora, Victoria, Australia; 4 La Trobe University, Bundoora, Victoria, Australia; 5 Facultad de Agronomia, Universidad de Buenos Aires, Buenos Aires, Argentina; Agriculture and Agri-Food Canada, Canada

## Abstract

**Background:**

*Paspalum dilatatum* Poir. (common name dallisgrass) is a native grass species of South America, with special relevance to dairy and red meat production. *P. dilatatum* exhibits higher forage quality than other C4 forage grasses and is tolerant to frost and water stress. This species is predominantly cultivated in an apomictic monoculture, with an inherent high risk that biotic and abiotic stresses could potentially devastate productivity. Therefore, advanced breeding strategies that characterise and use available genetic diversity, or assess germplasm collections effectively are required to deliver advanced cultivars for production systems. However, there are limited genomic resources available for this forage grass species.

**Results:**

Transcriptome sequencing using second-generation sequencing platforms has been employed using pooled RNA from different tissues (stems, roots, leaves and inflorescences) at the final reproductive stage of *P. dilatatum* cultivar Primo. A total of 324,695 sequence reads were obtained, corresponding to c. 102 Mbp. The sequences were assembled, generating 20,169 contigs of a combined length of 9,336,138 nucleotides. The contigs were BLAST analysed against the fully sequenced grass species of *Oryza sativa* subsp. *japonica*, *Brachypodium distachyon*, the closely related *Sorghum bicolor* and foxtail millet (*Setaria italica*) genomes as well as against the UniRef 90 protein database allowing a comprehensive gene ontology analysis to be performed. The contigs generated from the transcript sequencing were also analysed for the presence of simple sequence repeats (SSRs). A total of 2,339 SSR motifs were identified within 1,989 contigs and corresponding primer pairs were designed. Empirical validation of a cohort of 96 SSRs was performed, with 34% being polymorphic between sexual and apomictic biotypes.

**Conclusions:**

The development of genetic and genomic resources for *P. dilatatum* will contribute to gene discovery and expression studies. Association of gene function with agronomic traits will significantly enable molecular breeding and advance germplasm enhancement.

## Introduction

*Paspalum dilatatum* Poir. (common name dallisgrass) is a highly productive C4 grass with a wide distribution within temperate-warm regions and a growing season from late spring to late summer. *P. dilatatum* belongs to the Panicoideae subfamily within the PACMAD clade (Panicoideae, Arundinoideae, Chloridoideae, Micrairoideae, Aristidoideae and Danthonioideae), which contains all C4 grasses. It is believed that the PACMAD clade separated from the lineage that generated the more widely studied Pooideae and Bambusoideae grasses some 40–50 million years ago (Mya) [1]. Recently the C4 grasses have attracted considerable interest for bioenergy production and several of the key species under investigation (*Miscanthus giganteus*; *Panicum virgatum*; *Pennisetum purpuretum*) belong to the Paniceae clade and have probably diverged from the *P. dilatatum* clade by only c. 20 Mya. *P. dilatatum* is native to South America with special relevance to dairy and beef production [2], [3] as its forage quality is higher than that of other C4 forage grasses [4], [5], [6], and it exhibits tolerance of frost [7], [8] and water stress [9], [10].

There are several sexual and apomictic cytotypes (4×, 5×, 6×, 7×) and also biotypes of *P. dilatatum* that have been identified due to their morphological and distributional differences [11]. The predominant biotype found has purple anthers and apomictic reproduction (2n = 50), which is widespread in South America and naturalised in other parts of the world including China, USA, India, Japan, New Zealand and South Africa [12]. The sexual biotypes are typically characterised by yellow anthers. The genome constitution of the different ploidy levels has been described as IIJJ for tetraploids and IIJJX for the more common pentaploid biotype [13], [14]. The I genome has been identified in the diploid *P. intermedium* [15], which is believed to be the progenitor of the original genome donor. Cytogenic and phylogenic studies suggest that the donor of the J genome is believed to be *P. juergensii* [16]. The donor of the X genome is unknown, though the apomictic character is thought to have originated from this genome. The genome size has been estimated in different biotypes of *P. dilatatum*, with the tetraploid and pentaploid genome contents being 2.43 pg/2C and 2.96 pg/2C per nucleus, respectively [11].

*P. dilatatum* is likely to have more widespread use in livestock grazing systems under predicted climate change scenarios due to its superior drought tolerance and water use efficiency, in comparison to commonly-used C3 grasses. However, conventional breeding methods for improvement of *P. dilatatum* are commonly limited by its asexual reproduction system [17]. In apomictic reproduction, seeds are produced without fertilisation, generating uniform progeny that are genetically identical to their parents, restricting the genetic variation available for conventional breeding programs. As a result of this apomictic character, large clonal monocultures are often maintained in production zones, potentially jeopardising the feed-base of the grazing industries if adverse biotic or abiotic pressures emerge. The common type of *P. dilatatum* (apomictic and pentaploid) has a documented susceptibility to *Claviceps paspali*, an ergot fungus, which is the causal agent of “*Paspalum* staggers” in cattle that graze infected pasture [18]. To address these issues, advanced novel breeding strategies that employ genetic diversity must be developed and enabled to ensure the production of advanced cultivars to generate options for production systems.

Previous efforts to improve dallisgrass through breeding include the use of somaclonal variation [19], [20], [21] and interspecific hybridisation [22], [23]. Advanced molecular breeding techniques may be applied to the sexual cytotype or potentially to hybrids produced with the apomictic biotypes as pollen donors or with other closely related *Paspalum* species. Molecular markers are a valuable tool to identify and quantify genetic diversity within and between species, populations and available germplasm. These genetic resources can also link genotypes with economically important traits and assist breeders with germplasm selection and potential crossing schemes [24].

Genomic and transcriptomic resources for *P. dilatatum* are limited to only 77 nucleotide and 37 protein sequences. More broadly in the genus *Paspalum* the number of nucleotide sequences that are available is only 3,472 (NCBI 05/11/2013). Therefore, there is a significant need to increase the genomic resources for this species. Recently, second generation sequencing technology has been applied in a wide variety of areas including whole genome resequencing and *de novo* sequencing; as well as transcriptome analysis [25]. The Roche 454 Life Sciences GS FLX sequencing platform has been widely used for transcriptomic studies in non-model organisms (a full list of publications is directly available from http://454.com/publications/index.asp) [26], [27], [28]. Complete genome sequences have been generated for only a few grasses, as despite the advances made, undertaking to produce a whole genome sequence of a higher plant is still a significant task that requires considerable resources. The genome sequences of particular interest when studying *Paspalum* species are *Sorghum bicolor* L. [29] and foxtail millet, *Setaria italica* [30]. Both of these species are likely to have diverged from the *Paspalum* clade c. 10 Mya and represent two highly relevant genomes for enhancing any generated data. These two related reference genomes could assist in sequence annotation of *P. dilatatum*. Due to the complexities of undertaking complete sequencing of genomes from higher plants, transcriptomic sequencing of *P. dilatatum* would generate an initial resource of gene sequences as well as identifying valuable molecular markers such as EST-Simple Sequence Repeats (SSRs) for molecular breeding to improve the productivity and quality of this forage species.

The use of the second-generation sequencing technology is a cost-effective method to obtain large-scale EST sequences of non-model species. For the generation of a resource of largely full length sequences derived from transcriptomics, the longer read length of the GS FLX Titanium chemistry is ideally suited to generate such a data set. The GS FLX platform is the most widely used technology for the identification of SSR motifs and the development of molecular markers in plants [31]. EST-derived simple sequence repeat (SSR) molecular markers have the advantage that by definition they are genic and therefore can be potentially associated with functional regions, avoiding the non-coding and repetitive DNA in species with large genomes. EST-based molecular markers might also be linked to genes controlling important agronomic traits that could assist breeding programs and have been used to improve crop species [32], [33], [34]. Furthermore, due to their conserved location, EST-SSR can be transferable between related species [34]. A recent study has developed a cohort of 17 genomic SSR markers for *Paspalum atratum* Swallen and *Paspalum notatum* Flüggé. These SSRs were evaluated broadly across the Paspalum genus allowing species variability to be distinguished [35].

This study provides the first *de novo* assembly and annotation of a cDNA transcriptome dataset for *P. dilatatum*, derived from multiple tissues of a single plant at the reproductive stage of its life cycle. An extensive collection of EST-SSR molecular markers have subsequently been identified and a subset validated. This has generated a valuable resource of gene sequences and molecular markers for future breeding programs for this important forage species.

## Materials and Methods

### Plant material and growth conditions

*P. dilatatum* cv. Primo, a tetraploid sexually-reproductive cultivar and *P. dilatatum* cv. Relincho, a pentaploid apomictic cultivar, were kindly provided by Gustavo Schrauf (University of Buenos Aires, Argentina) and used as source plant material for all experiments. Individual plants were grown under glasshouse conditions (21°C, 14 h photoperiod/14°C, 10 h dark period). Plant tissues were harvested for RNA isolation from a single clone of *P. dilatatum* cv. Primo at the late reproductive stage, from leaves, stems, roots and mature inflorescences.

### RNA extraction and cDNA synthesis

Total RNA was isolated using RNeasy® Plant Mini Kit (Qiagen, Hilden, Germany) following manufacturer's instructions. RNA integrity was visually assessed on a 1% (w/v) agarose gel stained with SYBR® Safe DNA gel stain (Life Technologies, Carlsbad, USA), before proceeding with cDNA synthesis. A total of 250 ng of total RNA from each RNA extraction was combined to provide a total of 1 µg for use in cDNA synthesis using the SMART™ cDNA Synthesis kit (Clontech Laboratories, Mountain View, USA), according to the manufacturer's instructions. Messenger RNA (mRNA) was reverse-transcribed using SMARTScribe™ Reverse Transcriptase and SMART IV™ oligonucleotides. A modified poly-T primer sequence (5′-AAGCAGTGGTATCAACGCAGAGTCGCAGTCGGTACTTTTTTCTTTTTTV-3′) was used instead of the CDS-II primer to reduce sequencing problems associated with the poly-A tails [36].

### EST sequence generation

Approximately 5 µg of the amplified cDNA library was sheared by nebulisation at 200 kPa for 2 min. The concentration and integrity of the nebulised cDNA sample was determined using a Bioanalyzer 2100, with a DNA 12000 Labchip (Agilent, Santa Clara, USA) according to manufacturer's instructions. The GS-FLX titanium shotgun sequence library was then prepared following the manufacturer's instructions (Roche Diagnostics, Basel, Switzerland). The quality of sequencing library was then assessed using a Bioanalyzer 2100, fitted with an RNA 6000 Pico Lab Chip (Agilent) according to the manufacturer's instructions. Quantification of the sequence library was performed using quantitative real-time PCR. Emulsions were prepared using the Large Volume Emulsion PCR Kit (Roche Diagnostics). Finally, enriched beads were loaded onto one half of a picotitre plate for sequencing following the manufacturer's protocol.

### Assembly and annotation

Primary sequence output has been deposited in the sequence read archive of GenBank (accession number SRR1012849). Adapter and poly A sequences were trimmed from all sequence reads and the sequences were then *de novo* assembled, both procedures were performed using Newbler v 2.0.01.14 software (Roche Diagnostics). The coding sequences (CDS) from *Oryza sativa japonica* v6.1, *Sorghum bicolour* v1.0, *Brachypodium distachyon* v1.0 and *Setaria italica* v2.1 were downloaded from the GenBank and Phytozome websites (www.phytozome.net) and converted into a local custom BLAST database. The *P. dilatatum* sequence contigs were then used as the query sequences in a BLASTn analysis with a threshold E-value of 10^−10^. Only the most significant match was recorded for each sequence contig.

The UniRef 90 (www.ebi.ac.uk/uniref/) protein database was downloaded and converted into a custom BLAST database. The *P. dilatatum* sequence contigs were used as queries using BLASTx analysis with a threshold E-value of 10^−10^ and the most significant matches were recorded. Gene Ontology (GO) classification was assigned for each contig that matched the *Oryza sativa* protein database.This was performed by selecting the rice gene IDs that were identified from the initial BLASTn analysis and extracting the GO terms for that subset of genes.

### EST-SSR Identification and validation

Identification of di-, tri-, tetra-, penta- and hexanucleotide SSR loci, with minimum repeat numbers of 6, 4, 3, 3, and 3, respectively, and design of corresponding primers was performed using Batch Primer 3 software (http://probes.pw.usda.gov/batchprimer3/). The parameters for primer design were as follows: primer length range = 18 to 23 nucleotides (21 optimum); optimum annealing temperature = 55°C; GC content 30–70% (50% optimum).

A collection of 96 primer pairs were synthesized for empirical testing. Forward primers were synthesised with the addition of the M13 sequence to the 5′ end, to enable fluorescent tail addition through the PCR amplification process [37]. Genomic DNA was extracted from fresh leaf tissue from a collection of 49 genotypes from *P. diltatum* cv. Primo and one sample from *P. dilatatum* cv. Relincho using the DNeasy® 96 Plant Kit (QIAGEN) according to the manufacturers' instructions. PCR reactions were performed in a 12 µL volume containing Immolase PCR buffer, 1.5 mM MgCl_2_, 200 µM dNTPs, 0.016 µM of M13 FAM universal primer, 0.041 µM of M13 tailed forward primer and 0.16 µM of reverse primer, 0.25 units of Immolase DNA polymerase (Bioline, London, UK) and approximately 10 ng of *P. dilatatum* genomic DNA. Cycling conditions were as follows: 95°C for 10 min, followed by 35 cycles of 95°C for 30 sec, 55°C for 30 sec and 72°C for 30 sec and a final elongation step of 72°C for 10 min, performed using an Applied Biosystems Geneamp 9700 thermal cycler (Life Technologies). Amplification products were diluted 40-fold with the addition of water, and 2 µL of product was then combined with 8.95 µL of Hi-Di™ formamide (Life Technologies) and 0.05 µL of GeneScan™ 500 LIZ® Size Standard (Life Technologies). PCR products were processed using an ABI3730xl DNA analyser and amplification product sizes were determined using GeneMapper® v 3.7 software (Life Technologies).

## Results

### EST sequencing and *de novo* assembly

After initial sequence base calling and quality assessment had been performed, a total of 324,695 sequence reads, generating a total of 102,539,862 nucleotides were obtained. The average and modal sequence lengths were 338 and 430 nucleotides, respectively.

**Figure 1:**
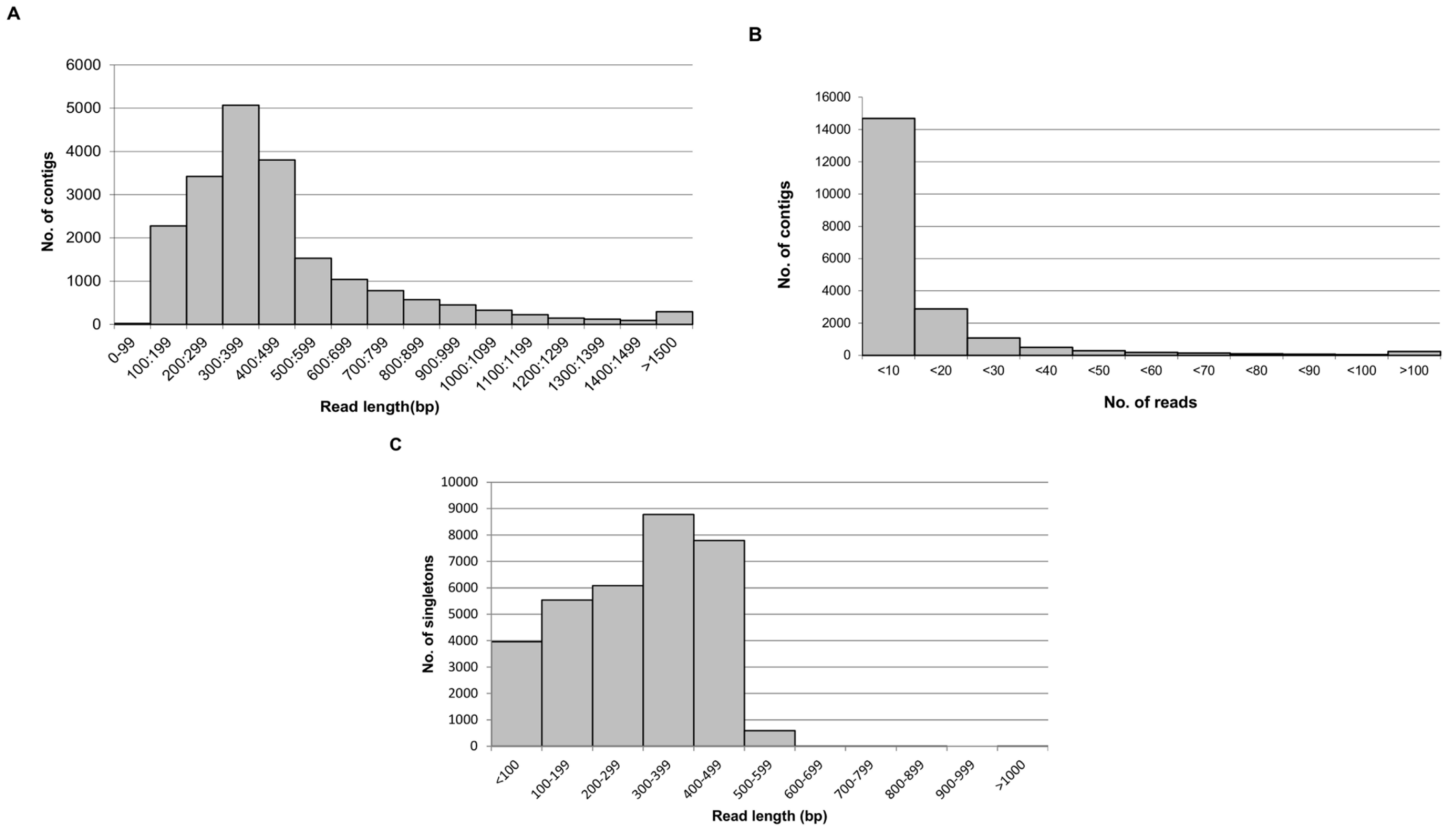
Frequency histograms showing the distribution of contigs compared to sequence read length (A), the distribution of the number of reads per contig (B), and distribution of singletons compared to read length (bp) (C).

After further quality filtering by removing adapter and primer sequences, reads were *de novo* assembled resulting in 20,169 contigs of a combined length of 9,336,138 bp and 32,751 singletons (Fasta Files S1, S2). The contig length varied from 104 to 7,877 bp. The majority of the contigs varied in length from 200–499 bp (60.9%) with the largest proportion being 300–399 bp (25%; Figure 1A). A total of 5,580 contigs (25%) were identified with a length greater than 500 nucleotides. The majority of the contigs (72.8%) were derived from less than 10 reads (Figure 1B).

The minimum accepted sequence length from the unassembled singletons was 50 bp. The average sequence length of the singletons was 374 bp and the largest proportion of the singletons (26.7%) were between 300–399 bp in length and only 25.6% were longer than 400 bp. There were a total of 17 singleton reads (1.8%) that were longer than 600 bp and represent sequences with long repeats of single nucleotides (Figure 1C).

### Gene annotation

**Figure 2:**
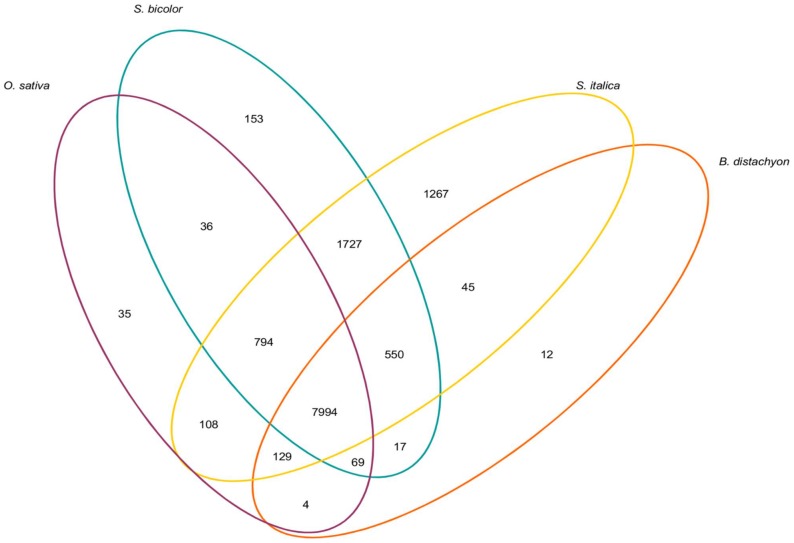
Sequence analysis of the generated *P. dilatatum* contigs compared to the reference grass genomes. The *P. dilatatum* contigs were compared using BLASTn with an e value threshold of 10^−10^ and significant matches were identified and totaled and presented as a Venn diagram.

The *P. dilatatum* sequence contig dataset was reciprocally analysed, using the BLASTn algorithm against the CDS data sets of the relevant grass species with whole genome sequences. These data sets were *Oryza sativa* subsp. *japonica* (v6.1), *Brachypodium distachyon* (v1 assembly), *Sorghum bicolor* (v1.0 release, Sbi1.4 gene set) and foxtail millet (*Setaria italica* v2.1). This analysis revealed that 45.4% of the *P. dilatatum* cDNA contigs matched *O. sativa* genes; 43.7% matched *B. distachyon* genes, 56.2% matched *S. bicolor* genes and 62.5% matched CDS sequences from the foxtail millet assembly (Figure 2 and Table S1). A total of 7,994 (39.6%) of the *P. dilatatum* contigs had significant BLASTn matches to sequences from all of the reference genomes, while 7,229 (35.8%) failed to find a significant match sequences from any of the reference genomes.

**Figure 3:**
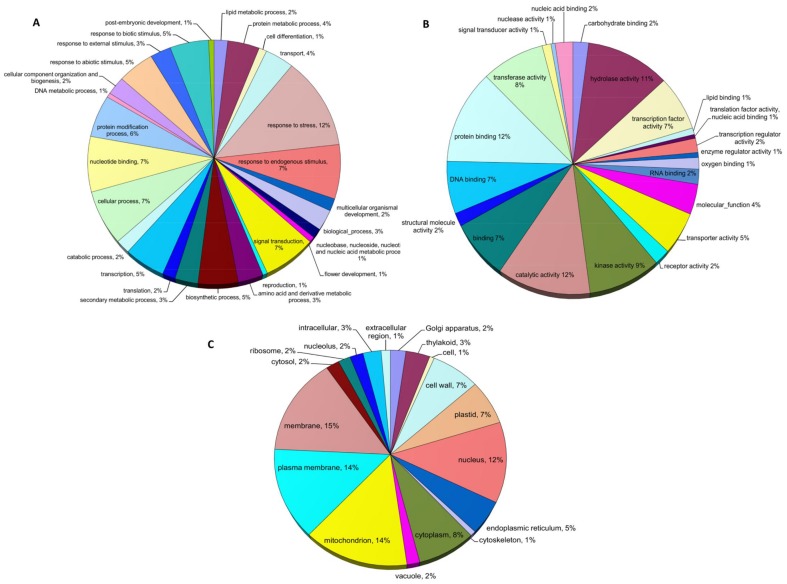
Assignment of putative function and gene ontology (GO) annotations to *P. dilatatum* sequence contigs based on Biological process classification (A), Molecular function (B) and Cellular components (C).

The contigs were compared against the UniRef 90 protein database using the BLASTx algorithm with an E-value cutoff of 10^−10^ to assign putative functional roles and annotations to the *P. dilatatum* trascriptome data set (Table S1). A total of 11,417 contigs (56.6%) identified a protein from the uniref data base. Gene ontology (GO) terms were also assigned for the subset of contigs that matched the rice genome. Unigenes were classified into three GO terms: biological process, molecular function and cellular component. A total of 76,093 gene counts and 104,741 annotation counts were assigned. The majority of assignments belonged to the biological process ontology (41.4%) followed by molecular function (34.1%) and cellular components (24.5%). Among the biological process category, response to stress (12%) was the most highly represented category, followed by response to endogenous stimulus (7%), nucleotide binding (7%), cellular process (7%) and signal transduction (7%). Other functional classifications were represented at proportions less than 5% of the total (Figure 3A). In the molecular function classification class, catalytic activity (12%), protein binding (12%), hydrolase activity (11%), kinase activity (9%) and transferase activity (8%) constituted the major categories (Figure 3B). The mitochondrion (14%), plasma membrane (14%) and membrane (15%) categories of the cellular component ontology contributed to the largest proportion of annotations followed by nucleus (12%), cytoplasm (8%) and plastid (7%) (Figure 3C).

### EST-SSR discovery and validation

**Figure 4:**
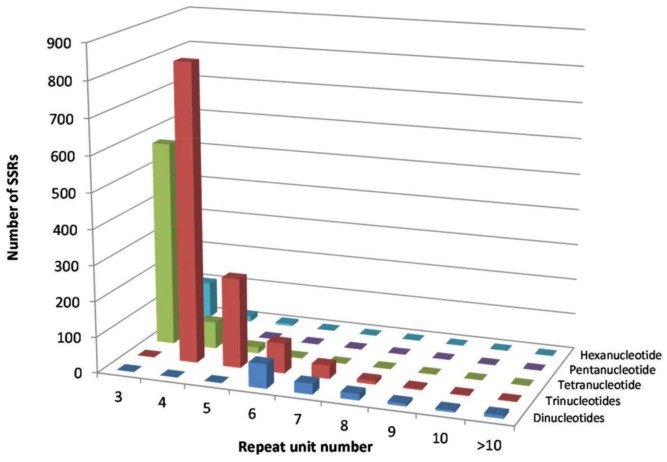
Distribution and frequency of different SSR repeat motifs identified from the *P. dilatatum* contig dataset.

The assembled contigs from the EST transcript data were chosen for SSR discovery and validation. Moderate length of sequence is required for SSR motif identification and reliable amplification following primer design. In addition to this, unique locus status for each SSR is desirable, which is best served through analysis of the assembled contigs to avoid partial sequences of ESTs. The 20,169 contigs were processed through batch Primer 3 software for motif identification and design. A total of 2,339 EST-simple sequence repeats (SSR) motifs (di-, tri-, tetra-, penta- or hexa-nucleotide) were identified from a total of 1,989 contigs (11.6%). The most frequent SSRs motifs in *P. dilatatum* transcriptome data set were tri-nucleotide repeats (Figure 4).

High quality primers were designed using BatchPrimer 3 software and a sub-set of 96 primer pairs were synthesised and used for empirical validation. All identified SSRs along with the designed primers are provided in table S2. Initial testing of the SSRs was performed on a single DNA sample from a distinct plant of the sexual tetraploid cultivar Primo and a DNA sample from the asexual apomictic cv. Relincho. Acceptable performance, defined as generation of distinct amplification products when resolved through capillary electrophoresis, was obtained for 64% of the SSRs that underwent initial validation. An additional collection of 48 plants from cv. Primo were screened with the selected SSR markers. The data from both rounds of SSR marker analysis was combined. A total of 32 SSR markers were able to identify variation between the sexual and apomictic cultivars, whilst the remaining markers only generated monomorphic marker profiles across all of the samples tested. Within these 32 SSR markers, a subset of eight identified polymorphic features within the samples from the cultivar Primo. The SSR markers typically generated fewer products from samples from cv. Primo than Relincho with median values of 2 and 3 respectively (Table 1).

**Table 1:** Details of SSR molecular markers from *P. dilatatum* validated as polymorphic within the current study.

SSR Id	Contig	Predicted Product size	SSR Motif	Motif Lenght	Forward Primer	Reverse Primer	Allele size range	Polymorphic in cv. Primo	Number of alleles in cv.Primo	Number of alleles in cv. Relincho
Pd0061	contig00359	128	AGGAGC	6	GCAGCTTCATCTCTATGTTTC	CTACATCAGGAGTAAGCGATG	139–149	No	1	4
Pd0228	contig01406	177	GGT	3	CTTCATTCCTTCAGATCCACT	TACACTGCATACACCGAAGA	153–205	No	4	5
Pd0240	contig01488	227	CCGA	4	AAACTGATGCTCCTCAACC	GTACAGCGGGTTGTTCTG	333–377	No	2	3
Pd0297	contig01864	157	CTGTGG	6	GTCCTCCTAGCTGTTGAAGAT	AGAAAAGACCTAAGCATGGAC	169–175	No	2	3
Pd0298	contig01883	126	CGC	3	CAAGATCCCTCCCTACTCC	ACATAGTCCTCCCCAACC	143–181	No	1	2
Pd0410	contig02637	165	TCG	3	GTCACCTCCTTATAGCCCTTA	CAAATCCAAAGACGATGC	180–187	No	1	2
Pd0630	contig04101	170	GCGA	4	AAGAGCTTCCCAACATCC	GATATTTCGGAGGAAGAAGAG	440–465	No	2	3
Pd0741	contig04909	181	TCT	3	GTGGATGGATGGATGGAT	TTCAGACACACTGCATCAAC	182–204	Yes	4	3
Pd0778	contig05150	184	GCGGAG	6	CTGAAGCAAGGGTAGGAGAT	ATCTCCTCCTGAGTTTGTACC	196–202	Yes	2	2
Pd0806	contig05407	157	GGA	3	AGGAGTGAGAGGAAGAACAAC	GATGACACAAGCAGTAGCAG	175–189	No	1	2
Pd1298	contig09623	154	GAG	3	TCCACCAAGAGCTCACAT	GCATCTGTCGTAGACTCGTAG	118–225	No	3	5
Pd1302	contig09667	183	CCTGTG	6	CAAGGTCATGTTCAACCTCTA	ACAGCACAAGAGCTAAAACC	175–236	No	2	3
Pd1357	contig10112	176	AGC	3	ATCATTTTTGCTGCTCTCTC	AGTATGGGAGGACGTTGTT	186–194	Yes	2	3
Pd1389	contig10385	159	CGG	3	CTCAGCTCCTAGTCGTCCTA	ACATTGCGGATAAATCTGAC	170–177	Yes	2	1
Pd1437	contig10830	150	GCA	3	CAACTTCACCAACAGCAAC	TGCTTATTCTTTCTGCGTTC	135–174	No	2	3
Pd1480	contig11038	167	CGG	3	ATCAGAAAGAGGGATTTTGC	AAATCAGGGCAAAGAGGTAG	182–185	No	1	2
Pd1488	contig11123	157	AAGAGG	6	CTCCTGGCTTCCTAGATTTAC	CAATTGAAACATGGTCTGC	173–189	Yes	2	2
Pd1505	contig11302	149	CGAGCA	6	ATCTTGCTCCATCACCATTA	CTTGTCTCCTACCTCACCAC	150–166	No	1	3
Pd1515	contig11456	160	CCG	3	AGTCCACTTTCACTTCCACTC	CTCTCCATCCTGCACCTC	171–179	No	2	4
Pd1533	contig11692	153	GGC	3	GGTCTTGAGGAGTGAGAGC	GAGAGAAAGCTAGGGTTTTGA	248–267	No	3	4
Pd1594	contig12224	160	GCA	3	TCAGTAGGACACACAACATCA	TAGTAGTTGGACGGATTTGAG	165–174	Yes	4	2
Pd1602	contig12343	122	GCG	3	ACTTCCGCTTCTTGTTGAT	ATCCAAGTCTAGGGTTCCAC	121–138	No	1	3
Pd1740	contig13647	148	AGCC	4	GGGATTATACAAATGGAGGAG	TGTTCGATTCTCATCAGTAGG	156–163	No	1	2
Pd1838	contig14705	162	TTTGTG	6	GAGATGTAATAGCGGTGTTTG	CTCAGCTCCACAGTCACAC	176–183	No	1	1
Pd1887	contig15149	140	TCA	3	GGACCAGCAGACACAAAC	CCACATCTTCACCTCTCTCTC	269–271	No	1	2
Pd1907	contig15377	139	CCG	3	ACAAGGCCAGAAGGGTTT	CAAAAGCAGTGGCAAGAG	150–153	No	1	1
Pd2162	contig18221	176	GCG	3	GTTGTACCTGATGGTGTTGTC	CCGATCAAGGGTTTCCTC	289–298	Yes	2	2
Pd2239	contig18901	148	CAT	3	GCTGCTGTTATTCTGTACCTG	CCTTCTCTTTCTCTCCAAGAC	160–166	Yes	2	2
Pd2251	contig19078	150	CGG	3	CTTCTGGTCGTAGGTGTAGTC	CTCCCCCTTCTTCACTCC	248–264	No	2	4
Pd2273	contig19311	155	GTCCCC	6	ACCGCCAAAAACCAAGAG	CTTCTTCTCCGCCTTCTC	166–177	No	1	4
Pd2337	contig20145	116	GCG	3	GGAAGGATTCTCTCCCTAGA	CTTCTTGTTGCCGAAGAAT	128–131	No	1	2
Pd2338	contig20158	153	GTG	3	GTCTGCGGCTCTTCTATTC	AAATGGACTAGAACCTCGAAC	168–203	No	2	4

## Discussion

Efforts to sequence grass genomes have increased considerably in recent years. The first crop species with a complete genome sequence was rice [38], followed by several other species within the Poaceae: sorghum [29], maize [39], brachypodium [40], and most recently foxtail millet [30]. In addition, there are large collections of EST sequences available from species within the Poaceae family (7,180,207 nucleotides ESTs, 20/07/2013) along with collections of next generation RNA sequence based data (1,791 entries in the SRA database 20/07/2013). However, the majority of these studies in recent times have focused on maize (>1,000 of the RNA based SRA entries for the Poaceae). An EST sequencing approach can provide a valuable collection of cDNA sequences and has been widely used for gene discovery and development of molecular markers, as it is a rapid, cost effective approach that can avoid well known problems associated with repetitive regions and large genome sizes in many non-model organisms where no prior data is available. Even though complete genome sequences have now been generated for some grasses, there are >11,000 recognised species within the Poaceae [41] so it is not surprising that genomic resources have been limited for most grass species, including *P. dilatatum*, prior to this study.

Next generation sequencing has been widely used within many crop species due to its low cost and greater sequence yield [42]. The availability of next generation sequencing technology enables large sequence data sets to be generated at modest costs. However, *de novo* sequencing of complex, often repetitive genomes, which are common in the Poaceae family, is not trivial. The characterization of genes with low expression levels or genes induced by specific environmental cues, or with spatially-restricted expression patterns can be challenging. Sequencing technologies that generate short reads (35–150 bp) such as the Illumina HiSeq systems are ideally suited to model organisms where reads can be mapped to reference genomes or transcriptome sequences. However, as the GS FLX platform can generate reads of >400 bp, it has been used for *de novo* annotation in non-model plants such as olive [42], [43], chestnut [44], ginseng [45],strawberry [46], bracken fern [47] and recently, in switchgrass [48].

This study reports the generation of genetic resources for *P. dilatatum* using the GS FLX Titanium platform. The EST-dataset generated in the current study provides a significant contribution towards the development of a resource in *P. dilatatum* that facilitates gene discovery and molecular marker development, for breeding programs. The study also provides a basis for future studies into gene expression under specific conditions with biotic or abiotic stresses. The GS FLX platform has a high error rate in homopolymeric regions (i.e., three or more consecutive identical DNA bases) caused by accumulated light intensity variance [49]. Therefore, a modified primer with an interrupted poly d(T) tail was used to avoid the limitations in sequence quality caused by the poly(A) tails of messenger RNA molecules. Similar approaches have been used in other transcriptome studies with significant benefits [45], [50].

The data generated was of high quality as the majority of the generated sequence reads assembled into contigs (74%) with an average contig length of 463 bp; which is also comparable to the average contig length obtained in previous studies (454 bp [51], 334 bp [49], 440 bp [45]. There were still however a significant proportion of sequence reads that were not assembled into contigs and indicate that further transcriptomic studies could benefit the sequence assembly and provide a comprehensive catalogue of full length cDNA sequences, and also unequivocally identify low quality uninformative reads. A small proportion of singleton reads were longer than 600 bp, a length that is outside of the standard read length of the GS FLX 454 platform using Titanium chemistry. These sequences contain long repeats of single nucleotides, which increases the defined length of the read passed the expected output size. It is likely that these sequence reads are an artefact of the sequencing process and are not derived from *P. dilatatum*, even though they have passed through the standard pipeline approach to sequence quality filtering.

Results of comparative sequence analysis reflects taxonomic relationships, with a high proportion of *P. dilatatum* reads matching sequences from other members of the Panicoideae subfamily within the PACMAD clade, (*Sorghum bicolor* L. and *Setaria italica*). From the recent comprehensive and robust assessment of molecular phylogeny for the grass species [41], it would appear that the closest taxonomic relationship is between *P. dilatatum* and *Sorghum bicolor* L. However, this taxonomic relationship is not mirrored by the comparative sequence analysis performed in this study, where the highest number of matching sequence reads was obtained from comparisons to *Setaria italica*. This could indicate gene specific evolution that *S. bicolour* has undergone in isolation or potentially some differences in genome assembly and annotation between the two reference genomes. By undertaking the comparative genetic analysis within this study a significant description of the generated cDNA sequences has been attempted. Whilst this study has not focussed on single genes, it is anticipated that the resources generated, the gene ontology, protein as well as the reference genome analysis, will provide assistance to trait specific studies in the future.

Despite being described in plant species since 1992 [52], SSR markers are still widely used for many applications, including population genetics and marker assisted breeding due to their codominant and highly polymorphic nature [53]. EST-SSRs have previously been associated with agronomically important traits in many species including cotton and maize [54], [55]. *De novo* development of a large cohort of SSRs was previously a costly and time-consuming process [56], [57]. However, SSRs can now be rapidly identified in EST databases generated by sequencing of transcriptomes. EST-SSRs have an enhanced cross-species transferability and efficiency of amplification in comparison with SSRs located in non-transcribed regions [58], [59], which could derive significant benefits for the c. 400 other *Paspalum* species [60]. A small cohort of genomic derived SSR markers were recently developed for *P. atratum* Swallen and *P. notatum* Flüggé and successfully transferred in 35 Paspalum species [35]. The use of SSRs across related species has been previously demonstrated in the Poaceae family: wheat EST-derived SSRs were transferable to eight related species [61] and to important crops including maize, barley and rice [62]. Barley (*Hordeum vulgare*) EST-derived SSRs were transferable to *H. chilense* lines and showed polymorphism [63]. The amplification of multiple products from tetraploid genotypes of *P. dilatatum* and amplification of even more products from pentaploid genotype in this study indicates that SSR markers are able to detect loci within the I, J and X genomes of *P. dilatatum*.

The frequency of EST- SSRs detected is typically influenced by the DNA template used, the criteria for defining SSRs, and the software and parameters used for identifying SSRs [64], [65], [66]. In this study, the frequency of trinucleotide repeat units was predominant, followed by tetra-, penta-, di- and hexanucleotide repeat units. The high frequency of trinucleotide repeat units is consistent with results previously reported in numerous plant species [67], [68], [69], [70], [71], [72]. Polymorphism in trinucleotide repeats in coding regions of a transcript would be more tolerated as they would not cause shift in the amino acid translation [73].

Conventional plant breeding is a costly and time consuming approach that could be enhanced with molecular marker based system to facilitate the selection of desirable agronomic traits. In apomictic plants, novel approaches for interspecies hybridization must be employed to increase variation and effectively use available germplasm resources. Critically, the selection of germplasm for breeding would benefit from prior genomic characterisation to make informed decisions for cultivar development. As an example the cultivar Primo, used in this study, is characterized with high potential forage production, but was developed based on an interspecific hybridisation and backcross strategy to incorporate *C. paspali* resistance from *P. urvillei* (also a sexual tetraploid *Paspalum* species with a genomic constitution of IIJJ) into *P. dilatatum* [74]. The application and screening of a modest subset of the discovered SSR markers across the germplasm detailed in this study, has already demonstrated that these SSR are functional across both the sexual and apomictic biotypes of *P. dilatatum*. However, the low level of SSR marker variance that was identified in this study from multiple samples of Primo could potentially be accounted for by the breeding history of the cultivar, which suggests that the value of the markers may be more accurately assessed by screening a more diverse collection of *Paspalum* germplasm.

## Conclusions

This study generated a substantial genomic EST-derived resource for the forage grass *Paspalum dilatatum* represented by 20,167 contigs, of which 12,940 have been sequence annotated. Furthermore, a collection of 2,339 SSR primer pairs have been designed and a subset validated for molecular breeding within the cultivated germplasm and can be assessed for efficiency across the genus *Paspalum*, dramatically advancing the available resources for this important forage grass species.

## Supporting Information

Fasta File S1 **Fasta file of generated contigs from the assembly process.**
(TXT)

Fasta File S2 **Fasta file of singletons remaining from the assembly process.**
(TXT)

Table S1 **Details and results from the BLAST analysis between the** ***P. dilatatum*** **contigs, and the reference CDS genomes and the UniRef 90 database.** Data is presented in tabular format.
(XLSX)

Table S2 **Details of all 2,339 SSR molecular markers identified within the study.**
(XLS)
